# A Meta-Analysis of Influencing Factors for Reinfection of Hand, Foot and Mouth Disease in China, Based on Adjusted Effect Estimates

**DOI:** 10.3390/pathogens15010050

**Published:** 2026-01-02

**Authors:** Anmin Ge, Weihong Cui, Siyu Qu, Ning Wang, Wenhua Zhang, Lili Wei, Shuqin Zhou, Quanman Hu, Liquan Zhang, Shuaiyin Chen

**Affiliations:** 1 College of Public Health, Zhengzhou University, Zhengzhou 450001, China; 2 Penglai Center for Disease Control and Prevention, Yantai 265600, China; 3 Department of Medicine, Shandong College of Traditional Chinese Medicine, Yantai 264199, China

**Keywords:** hand, foot and mouth disease, reinfection, influencing factors, meta-analysis, adjusted estimates

## Abstract

**Background:** Numerous studies have reported on the epidemiology of hand, foot and mouth disease (HFMD) reinfection and its potential influencing factors; however, findings regarding reinfection rates as well as determinants such as gender, age, residence, and pathogens remain inconsistent. Due to this heterogeneity in reported outcomes, a comprehensive systematic review and meta-analysis are warranted to consolidate existing evidence. **Methods:** Effect estimates were expressed as reinfection rates, odds ratio (OR)/hazard ratio (HR) and 95% confidence intervals (CI). When necessary, data were converted to ensure consistency across comparison groups. **Results:** A thorough search was carried out using the predetermined literature retrieval approach across the PubMed, Web of Science, and Embase databases. Finally, 9 articles met the inclusion criteria and were included in this study. The results indicated that the overall reinfection rate for HFMD was 4.1% (95% CI: 2.0–6.2%). Males compared to females (overall effect = 1.256, 95% CI: 1.176–1.341), younger compared to older children (overall effect = 2.972, 95% CI: 1.512–5.843), scattered children compared to students (overall effect: 4.017, 95% CI: 1.560–10.344), and enterovirus 71 (EV71) compared to non-EV71 enteroviruses (overall effect = 0.71, 95% CI: 0.59–0.86) were associated with the HFMD reinfection. **Conclusions:** The overall HFMD reinfection rate was 4.1% (95% CI: 2.0–6.2%). Male, younger age, kindergarten children, and infection with non-EV71 enteroviruses (compared to EV71), were identified as significant risk factors for recurrent HFMD. Targeted intervention strategies should be developed for these high-risk populations to effectively reduce the incidence of reinfection.

## 1. Introduction

Hand, foot and mouth disease (HFMD) is a common acute infectious disease that primarily affects infants and children under five years of age [1,2]. It is typically characterized by fever, rash and herpes on the hands, feet, mouth, and buttocks [3]. Most cases are mild and self-limiting, while in a small proportion of children, the disease may rapidly progress within a short timeframe, leading to severe complications involving the nervous or cardiopulmonary systems—such as aseptic meningitis, neurogenic pulmonary edema, and myocarditis—and in some instances may result in death [4,5]. Typically, the primary causative pathogens of HFMD were enterovirus 71 (EV71) and coxsackievirus A16 (CV-A16). In recent years, there has been a notable shift in epidemiological trends, with coxsackievirus A6 (CV-A6) emerging as a predominant pathogen [6,7]. Innate immunity acts as the primary line of defense for the host against enterovirus invasion. It restricts viral replication and allows time for the subsequent adaptive immune response, which is essential for the host to eliminate enteroviruses. Nevertheless, certain enteroviruses, such as EV71, have developed distinct proteases like 2Cpro and employ long non-coding RNA to evade the host’s innate immune system surveillance. As a result, they can resist the host’s antiviral response and then replicate in large amounts, leading to the progression of HFMD [8,9].

Since the 1990s, HFMD has been persistently spreading across the Asia–Pacific region. In 1998, a large-scale outbreak occurred in Taiwan, resulting in 405 severe cases and 78 deaths [10]. In 2008, during the EV71-associated HFMD outbreaks reported in Fuyang, China, 353 cases (5.8%) were classified as severe, with 22 fatalities (0.36%) [11]. Due to its remarkable public health impact, HFMD was designated as a legally notifiable Class C infectious disease in China. In addition, countries across other regions, including Malaysia [12], Vietnam [13], and South Korea [14], have also reported outbreaks of HFMD. Notably, the economic burden associated with this disease is substantial. Most clustered outbreaks occur in schools and kindergartens, where the severity of illness and duration of treatment significantly contribute to overall costs. Moreover, due to the high volume of mild cases, these cases constitute the primary driver of the economic burden [15]. The considerable economic and social burden posed by HFMD has thus become an unavoidable public health concern.

Since the introduction of the EV71 vaccine in China in 2016, real-world evidence has demonstrated that two doses effectively reduce both the incidence of EV71-associated HFMD and the occurrence of severe HFMD cases caused by EV71 [16]. While the vaccine induces a sustained antibody response lasting more than two years, it does not confer lifelong immunity, leaving children susceptible to reinfection [17]. Furthermore, the vaccine offers no cross-protection against HFMD caused by non-EV71 pathogens. Given the extensive diversity of enteroviruses responsible for the disease, the absence of cross-immunity among distinct serotypes and subtypes [18], and the rapid decrease in maternal antibody titers against enteroviruses like EV71 and CVA16 in newborns, the quantity of susceptible individuals within the population is steadily rising, rendering reinfection an inevitable outcome [19,20].

Numerous studies have reported on the epidemiology of HFMD reinfection and its potential influencing factors; however, findings regarding reinfection rates as well as determinants such as gender, age, residence, and pathogens remain inconsistent [21,22,23]. Due to this heterogeneity in reported outcomes, a comprehensive systematic review and meta-analysis are warranted to consolidate existing evidence. Therefore, this study synthesizes data from published literature across multiple databases on the reinfection rate of HFMD and its associated risk factors, aiming to identify high-risk populations and inform targeted prevention strategies.

## 2. Materials and Methods

### 2.1. Literature Search Strategy

A thorough search was carried out across PubMed, Web of Science, and Embase databases to retrieve published literature until 24 October 2025. A comprehensive search strategy is employed, incorporating the following terms: (“HFMD” OR Hand Foot Mouth Disease). and (Recurrence OR “Repeated infection” OR “Repeated onset” OR “Multiple infection”), The specific search strategies and results for each database are presented in Table S1.

An extensive search was performed to locate pertinent reviews and meta-analyses associated with the articles incorporated in this study, ensuring that no overlapping or redundant publications existed. Following this, the titles and abstracts of the identified studies were reviewed to determine eligibility before acquiring their full texts.

### 2.2. The Criteria for Inclusion and Exclusion

The criteria for inclusion were as stated below: (1) The study subjects are patients with recurrent HFMD. (2) The study needs to report the incidence rate of recurrent HFMD. (3) The study needs to clearly state the required data: odds ratio (OR)/hazard ratio (HR) and 95% confidence intervals (CI) of different influencing factors. (4) The included studies were observational studies without any intervention measures.

The exclusion criteria were as follows: (1) reviews, errata and comments; (2) studies whose themes were not related to recurrent HFMD; (3) studies with unclear data reporting, making it impossible to extract data.

### 2.3. Literature Screening and Data Extraction

This study was carried out following the PRISMA 2020 statement guidelines (Table S2), and the register ID is CRD420251179269 [24]. The screening of literature obtained from database searches was performed independently by two reviewers, guided by pre-established inclusion and exclusion criteria. Disagreements were resolved through consultation with a third author. Study quality was assessed using the Newcastle-Ottawa Scale (NOS): studies scoring ≥ 7 were considered high quality, those scoring 5 to <7 moderate quality, and those scoring < 5 low quality [25]. Data extraction included key details such as first author, study location, study period, age range, population, measurement index, and reinfection information.

### 2.4. Satistical Analysis

Data analysis was conducted using STATA software (version 12.1; Stata Corp, College Station, TX, USA), and statistical significance was set at α = 0.05. Effect estimates were expressed as reinfection rates, OR, HR, and corresponding 95% CI. When necessary, data were converted to ensure consistency across comparison groups. Heterogeneity among studies was assessed using the I^2^ statistic. An I^2^ value greater than 50% or a *p*-value less than 0.05 was considered indicative of significant statistical heterogeneity. In such cases, a random-effects model was applied to account for both within- and between-study variability. If heterogeneity was not significant, a fixed-effects model was used to calculate the pooled overall effect [26]. Subgroup analyses were conducted to explore potential sources of heterogeneity, based on factors such as study period, age range, population, effect measures, adjustment for confounding variables, and other relevant study-level factors. Publication bias was evaluated through visual inspection of funnel plots and by conducting Egger’s and Begg’s test. Sensitivity analysis was carried out to examine the robustness and reliability of the overall findings.

## 3. Results

### 3.1. Study Selection and Documentation of Included Studies

**Figure 1:**
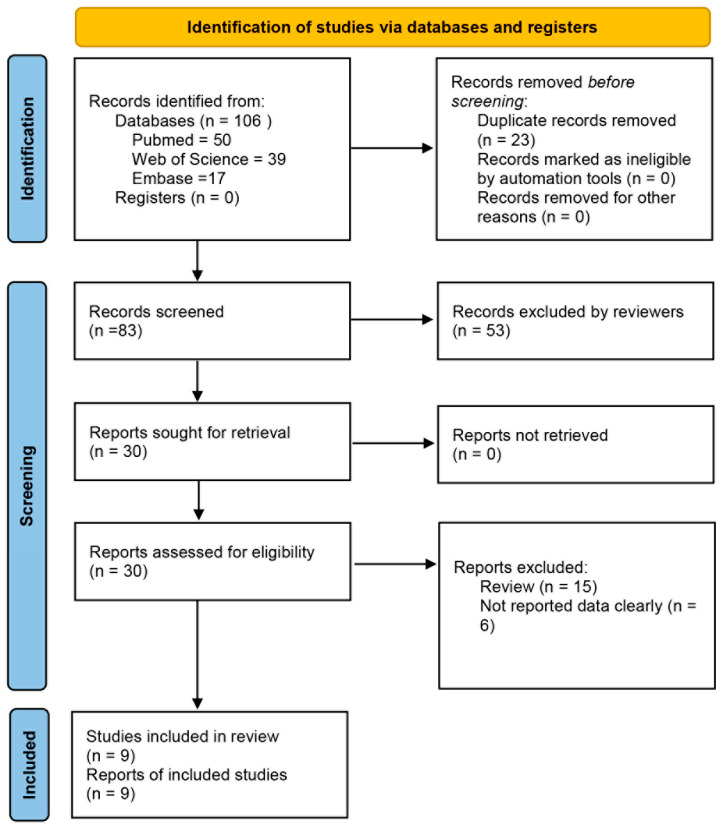
Flowchart showing the screening process for included articles.

A thorough search was carried out using the predetermined literature retrieval approach across the PubMed, Web of Science, and Embase databases, yielding a total of 106 articles. After duplicate removal, 83 articles were retained for further screening. Subsequent title and abstract screening led to the exclusion of 53 articles, leaving 30 articles for full-text assessment. Following detailed evaluation, 9 articles met the inclusion criteria and were included in this study (Figure 1). All 9 articles reported reinfection rates. The main factors associated with reinfection rates identified in these studies were gender (9 articles), age (9 articles), residence (7 articles), child type (6 articles), pathogens (5 articles), and disease severity (4 articles) [21,22,23,27,28,29,30,31,32].

Among the included studies, as shown in Table 1, the incidence of recurrent HFMD ranged from 2.02% to 7.00%. The study populations were primarily categorized into two age groups: ≤3 years and ≤14 years. Sample sizes varied from 1200 to 12,256,102, with study periods spanning from 2008 to 2023. The primary analytical methods employed were logistic regression models (reported as OR) and Cox proportional hazards models (reported as HR). The studies covered most regions of China. Study from other countries was excluded due to failure to meet the predefined inclusion criteria. All included studies had a NOS score of at least 6 points, indicating high methodological quality (Table S3).

### 3.2. Effect Estimates

**Figure 2:**
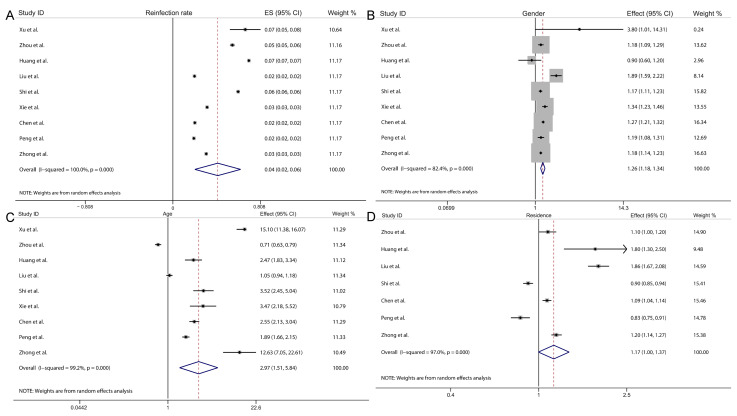
Forest plot showing the overall effect and 95% CI of reinfection rate (**A**), gender (**B**), age (**C**) and residence (**D**) [21,22,23,27,28,29,30,31,32].

**Figure 3:**
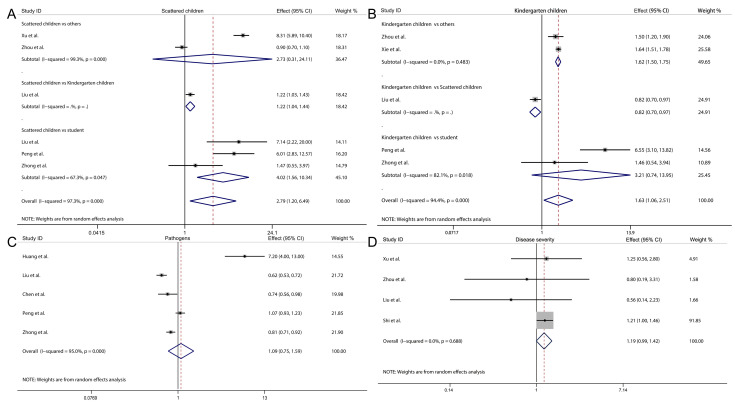
Forest plot showing the overall effect and 95% CI of scattered children (**A**), kindergarten children (**B**), pathogens (**C**) and disease severity (**D**) [21,22,23,27,28,29,30,31,32].

Based on the 9 studies included in this analysis, as illustrated in Figure 2 and Figure 3, we conducted a comprehensive assessment of overall effect estimates and 95% CI for factors including reinfection rate, gender, age, residence, child type, pathogens, and disease severity. The results indicated that the overall reinfection rate for HFMD was 4.1% (95% CI: 2.0–6.2%, I^2^ = 100%, *p* < 0.05, Figure 2A). Males were associated with a 25.6% higher risk of HFMD reinfection compared to females (overall effect = 1.256, 95% CI: 1.176–1.341, I^2^ = 82.4%, *p* < 0.05, Figure 2B). Younger children had a 197.2% increased reinfection risk relative to older children (overall effect = 2.972, 95% CI: 1.512–5.843, I^2^ = 99.2%, *p* < 0.05, Figure 2C). No statistically significant difference was observed in HFMD reinfection risk between rural and urban residents (overall effect = 1.169, 95% CI: 0.996–1.373, I^2^ = 97.0%, *p* < 0.05, Figure 2D).

Furthermore, when analyzing different categories of children, we found that scattered children had a significantly higher risk of HFMD recurrence compared to students (overall effect: 4.017, 95% CI: 1.560–10.344, I^2^ = 67.3%, *p* = 0.0470, Figure 3A), and kindergarten children also exhibited an increased risk relative to others (overall effect: 1.621, 95% CI: 1.499–1.752, I^2^ = 0.0%, *p* = 0.483, Figure 3B). However, no significant differences were observed in the risk of HFMD recurrence between scattered children and others, or between kindergarten children and students (Figure 3A,B). Finally, no significant association was found between HFMD recurrence and different pathogens of the disease (overall effect: 1.093, 95% CI: 0.749–1.595, I^2^ = 95.0%, *p* < 0.05, Figure 3C), nor between severe and non-severe cases (overall effect: 1.187, 95% CI: 0.993–1.419, I^2^ = 0.0%, *p* = 0.688, Figure 3D).

### 3.3. Subgroup Analysis

Our subgroup analysis was performed according to the basic characteristics of the included studies, specifically focusing on study period, age range, population, effect measures, and adjustment for confounding variables. Subgroup analyses were conducted separately for each factor. Notably, subgroup analysis was not carried out for factors with only a single category or level, as such only univariate indicators do not allow meaningful subgroup comparisons.

Regarding the reinfection rate, we performed subgroup analyses according to various factors. Notably, studies using the HR as the effect measure exhibited low heterogeneity (overall reinfection rate = 2.0%, 95% CI: 2.0–2.0%, I^2^ = 0.0%, *p* = 0.386; Figure S1D). For gender, it was found that the heterogeneity was relatively small among studies with study periods of 5–10 years: (overall effect = 1.20, 95% CI: 1.14–1.34, I^2^ = 62.7%, *p* = 0.030, Figure S2A); The heterogeneity among studies with age ≤ 3 years was relatively small: (overall effect = 1.29, 95% CI: 1.18–1.35, I^2^ = 14.6%, *p* = 0.030, Figure S2B); The heterogeneity among studies with effect measures = OR was relatively small: (overall effect = 1.22, 95% CI: 1.16–1.28, I^2^ = 68.7%, *p* = 0.030, Figure S2D).

For age, the heterogeneity among studies with participants age ≤ 3 years was relatively low (overall effect = 2.75, 95% CI: 2.21–3.57, I^2^ = 32.4%, *p* = 0.224; Figure S3B). Similarly, heterogeneity was low in studies conducted in large populations (overall effect = 2.53, 95% CI: 2.17–2.94, I^2^ = 0.0%, *p* = 0.865; Figure S3C). For residence, subgroup analysis did not identify a significant source of heterogeneity (Figure S4). Regarding pathogens, heterogeneity was substantially reduced among studies with a study period < 7 years (overall effect = 0.79, 95% CI: 0.70–0.90, I^2^ = 0.0%, *p* = 0.557; Figure S5A). Additionally, heterogeneity between studies comparing EV-71 and non-EV71 pathogens was minimal (overall effect = 0.71, 95% CI: 0.59–0.86, I^2^ = 0.0%, *p* = 0.032; Figure S5F).

### 3.4. Publication Bias and Sensitivity Analysis

**Figure 4:**
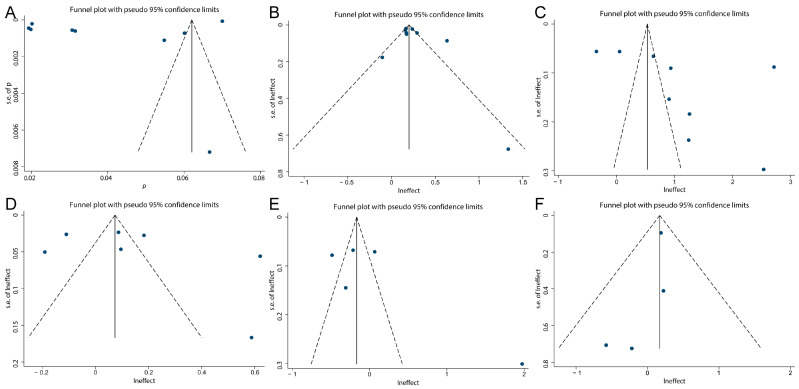
Funnel plot on reinfection rate (**A**), gender (**B**), age (**C**), residence (**D**), pathogens (**E**) and disease severity (**F**).

We conducted a publication bias assessment for the overall effect and 95% CI across various factors. Due to the limited number of included studies for child type subgroups, this analysis was not feasible for those categories. The evaluation employed funnel plots, Egger’s test, and Begg’s test as complementary methods. As shown in Figure 4, the funnel plot demonstrates symmetry for the overall effect and 95% CI regarding reinfection rate, gender, age, residence, pathogens, and disease severity, suggesting no evident publication bias. Consistently, the results of Egger’s and Begg’s tests show *p* values greater than 0.05 for all factors (Table S4), indicating no statistically significant small-study effects. Collectively, these findings suggest that the pooled estimates of reinfection rate and its associated influencing factors in this study are unlikely to be distorted by substantial publication bias.

**Figure 5:**
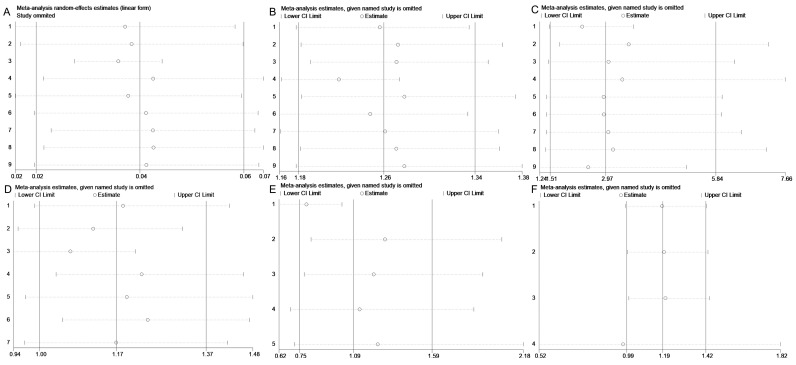
Sensitivity analysis on reinfection rate (**A**), gender (**B**), age (**C**), residence (**D**), pathogens (**E**) and disease severity (**F**).

Then, sensitivity analysis was conducted to assess the stability of the overall effect estimates of reinfection rate and its associated influencing factors. As shown in Figure 5, the overall effect and 95% CI for reinfection rate, gender, age, pathogens, and disease severity remained stable across the exclusion of individual studies, with consistent results observed when each included study was removed sequentially. However, the sensitivity analysis for residence demonstrated instability. Specifically, excluding the study by Shi et al. or the study by Peng et al., led to a notable shift in results compared to overall effect and 95% CI (Figure 5D). Additionally, we evaluated different pooling models and found discrepancies between the random-effects model (overall effect = 1.169, 95% CI: 0.996–1.373, I^2^ = 97.0%, *p* < 0.05) and the fixed-effect model (overall effect = 1.076, 95% CI: 1.049–1.104, I^2^ = 97.0%, *p* < 0.05), indicating model dependency in the overall effect and 95% CI.

## 4. Discussion

Children are particularly susceptible to HFMD due to their immature immune systems, which renders them vulnerable to enterovirus infections. However, antibodies generated against one subtype of enterovirus do not confer cross-protection against other subtypes, and immunity to the same subtype is not sustained indefinitely [33]. As a result, reinfection with HFMD is common. This study, based on a synthesis of nine published studies, found an overall reinfection rate of 4.1% (95% CI: 2.0–6.2%). The highest reported rate was 6.01% in Wuxi, China [30], while the lowest was 1.93% in Wuhan, China [22].

Regarding the factors influencing HFMD reinfections, nearly all studies indicate that the initial infection rate is higher in boys than in girls, a trend consistent with the first onset of HFMD [34]. One possible explanation for this gender disparity is that boys tend to be more physically active and behaviorally impulsive, thereby increasing their exposure to contaminated objects through contact with infected children [32]. Additionally, biological differences in immune responses between males and females may contribute, as enterovirus detection rates are generally higher in males than in females. With respect to age, younger children appear to be at greater risk of reinfection compared to older children. A cross-sectional study demonstrated that the seropositive rate of anti-EV71 antibodies in infants aged 0–6 months gradually declines, remains low from 7–11 months, and then begins to rise between 1 and 4 years of age. Among children aged 5–15 years, the specific anti-EV71 antibody positivity rate exceeds 80% [35]. This pattern is supported by the three studies in our review that stratified participants by age, each using ≥5 years as the reference group. All reported a decreasing risk of recurrent HFMD infection with increasing age among children under 5 years [21,30,32]. Furthermore, subgroup analyses revealed that studies focusing exclusively on children under 3 years of age reported a reduced reinfection risk compared to those including children up to 14 years of age. This may be attributed to the fact that although anti-EV71 antibody seropositivity increases in children under 3 years, the change is not statistically significant.

Compared with gender and age, no significant difference was observed in the risk of reinfection between individuals living in rural versus urban areas, although heterogeneity across studies remained relatively high. This inconsistency may be attributed to variations in geographic settings and levels of urbanization across the included studies. Some studies indicate a higher reinfection rate of HFMD among urban children, potentially due to high population density and substantial migrant populations in cities [30]. Conversely, other evidence suggests that reinfection rates are higher in rural areas, where urbanization has led to outmigration of young laborers, leaving behind children with limited hygiene awareness, compounded by inadequate healthcare resources, suboptimal living conditions, and lower educational attainment in rural regions [36]. Given the differing focus on child populations across studies, a pooled overall effect estimate was not calculated; instead, a stratified analysis was conducted. The results revealed that kindergarten children faced an increased risk of reinfection compared to other groups, and scattered children exhibited a higher risk than students. This may be explained by the crowded environments, confined spaces, and frequent interpersonal contact typical of kindergartens and daycare centers [37]. Additionally, scattered children are often younger and possess limited self-care abilities; their habitual finger-sucking behavior increases susceptibility to fecal-oral transmission, thereby facilitating reinfection [38].

For pathogens causing HFMD, there is no significant difference in the risk of repeated infection across different pathogen types. However, further subgroup analysis indicates that the risk of reinfection with EV71 is relatively lower compared to non-EV71 enteroviruses. This may be attributed to the higher environmental prevalence of EV71, leading to broader population immunity. Children initially infected with non-EV71 enteroviruses are more susceptible to subsequent infections [32]. Moreover, multiple studies consistently suggest that the likelihood of reinfection is not associated with disease severity during the initial episode. In cases of severe primary infection, which are predominantly caused by EV71, more intensive medical management may contribute to prolonged immune protection, thereby reducing the probability of reinfection [39]. Nevertheless, the current body of evidence remains limited, and additional well-designed studies are required to fully elucidate the mechanisms underlying reinfection dynamics.

However, this study has several limitations. First, the geographic scope of the included studies is confined to China. Although these studies cover a wide range of regions across the country, they do not extend to other countries. This may be attributed to the relatively high burden of HFMD in China, which has prompted greater attention from Chinese researchers toward recurrent infections. Second, for certain factors—such as disease severity—the number of eligible studies remains limited, thereby restricting the robustness of the conclusions that can be drawn. Finally, some of the included literature focuses on data collected prior to 2015. Given that the etiological profile of HFMD has evolved significantly in recent years, future research should prioritize more up-to-date studies to reflect current epidemiological trends.

## 5. Conclusions

This study conducted a comprehensive systematic review and meta-analysis to evaluate the reinfection rate of HFMD and its associated influencing factors. The results showed that the overall reinfection rate was 4.1% (95% CI: 2.0–6.2%). Furthermore, male, younger age, kindergarten children, and infection with non-EV71 enteroviruses (compared to EV71) were identified as significant risk factors for recurrent HFMD. Targeted intervention strategies should be developed for these high-risk populations to effectively reduce the incidence of reinfection.

## Figures and Tables

**Table 1:** Characteristics of the included studies of reinfection of HFMD.

Author	Study Location	Study Period	Age Range	Population	Measure Index	Adjust for Confounding Factors	Reinfection%	NOS Score
Xu et al. [27]	Wuhan, China	2020–2023	≤14	1200	OR	Partial	6.67	6
Zhou et al. [28]	Chongqing, China	2009–2023	≤14	42,032	OR	Partial	5.48	8
Huang et al. [23]	China	2008–2015	≤14	12,256,102	OR	Yes	7.00	9
Liu et al. [29]	Jingzhou, China	2009–2022	≤14	72,317	HR	Yes	1.99	7
Shi et al. [30]	Wuxi, China	2008–2016	≤14	107,677	OR	Yes	6.01	7
Xie et al. [31]	Fujian, China	2008–2010	≤3	82,929	OR	Yes	3.15	7
Chen et al. [32]	Anhui, China	2008–2013	≤3	444,076	OR	No	2.02	8
Peng et al. [22]	Wuhan, China	2008–2015	≤14	95,209	HR	Yes	1.93	7
Zhong et al. [21]	Guangzhou, China	2012–2017	≤14	95,710	OR	Yes	3.07	7

NOTE: HFMD: hand, foot and mouth disease; Partial: including adjusted or not for confounding factors.

## Data Availability

The original contributions presented in this study are included in the article/Supplementary Material. Further inquiries can be directed to the corresponding author.

## Supplementary Materials

The following supporting information can be downloaded at: https://www.mdpi.com/article/10.3390/pathogens15010050/s1, Figure S1: Subgroup analysis of overall effect and 95%CI for reinfection rate; Figure S2: Subgroup analysis of overall effect and 95%CI for sex; Figure S3: Subgroup analysis of overall effect and 95%CI for age; Figure S4: Subgroup analysis of overall effect and 95%CI for residence; Figure S5: Subgroup analysis of overall effect and 95%CI for pathogens; Table S1: Search strategy in different databases; Table S2: PRISMA 2020 Checklist; Table S3: NOS scores of included studies; Table S4: Begg’test of the overall RR and 95%CI of different air pollutants.
