# Supplementary material for: A Meta-Analysis of Influencing Factors for Reinfection of Hand, Foot and Mouth Disease in China, Based on Adjusted Effect Estimates
Source: Pathogens. 2026 Jan 2;15(1):50. doi: 10.3390/pathogens15010050 (PMC12844915; doi:10.3390/pathogens15010050)
Supplement: Supplementary file 1 [file pathogens-15-00050-s001.zip › Supplementary materials.pdf]

Supplementary materials

Figures and tables

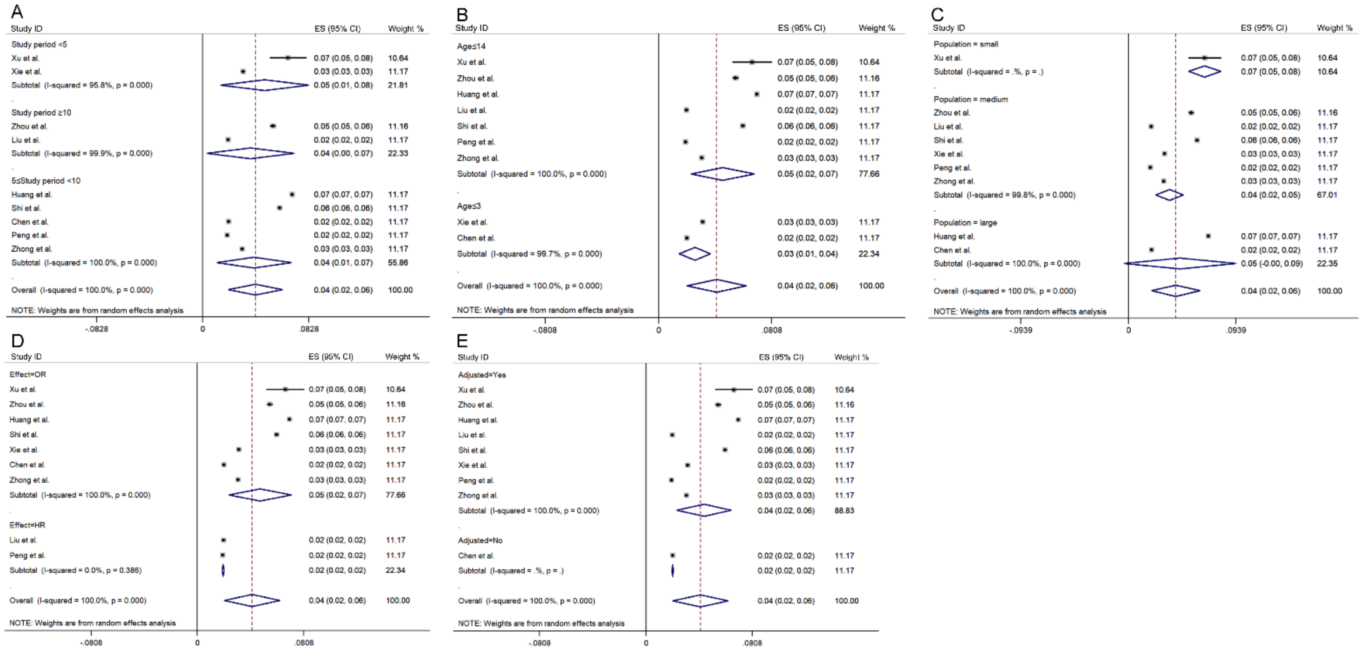

Figure S1 Subgroup analysis of overall effect and 95%CI for reinfection rate. A (study period); B (age range); C (population); D (effect measure); E (adjustment for confounding variables)

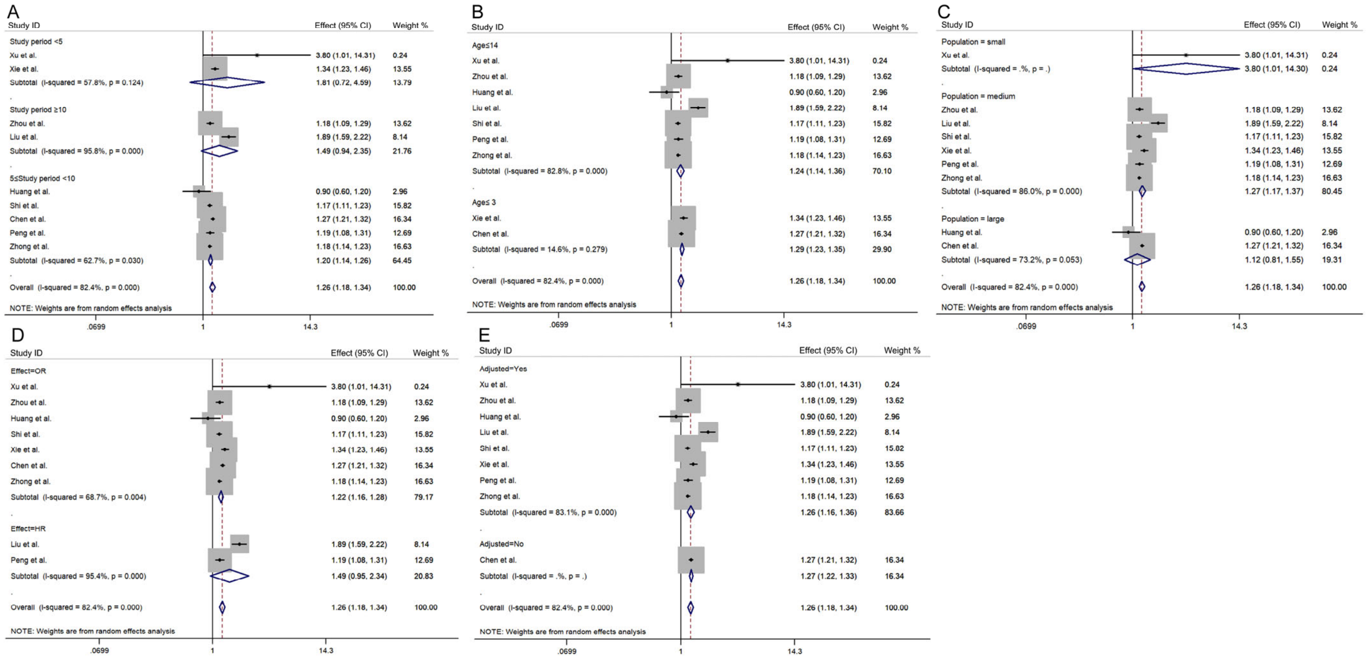

Figure S2 Subgroup analysis of overall effect and 95%CI for sex. A (study period); B (age range); C (population); D (effect measure); E (adjustment for confounding variables)

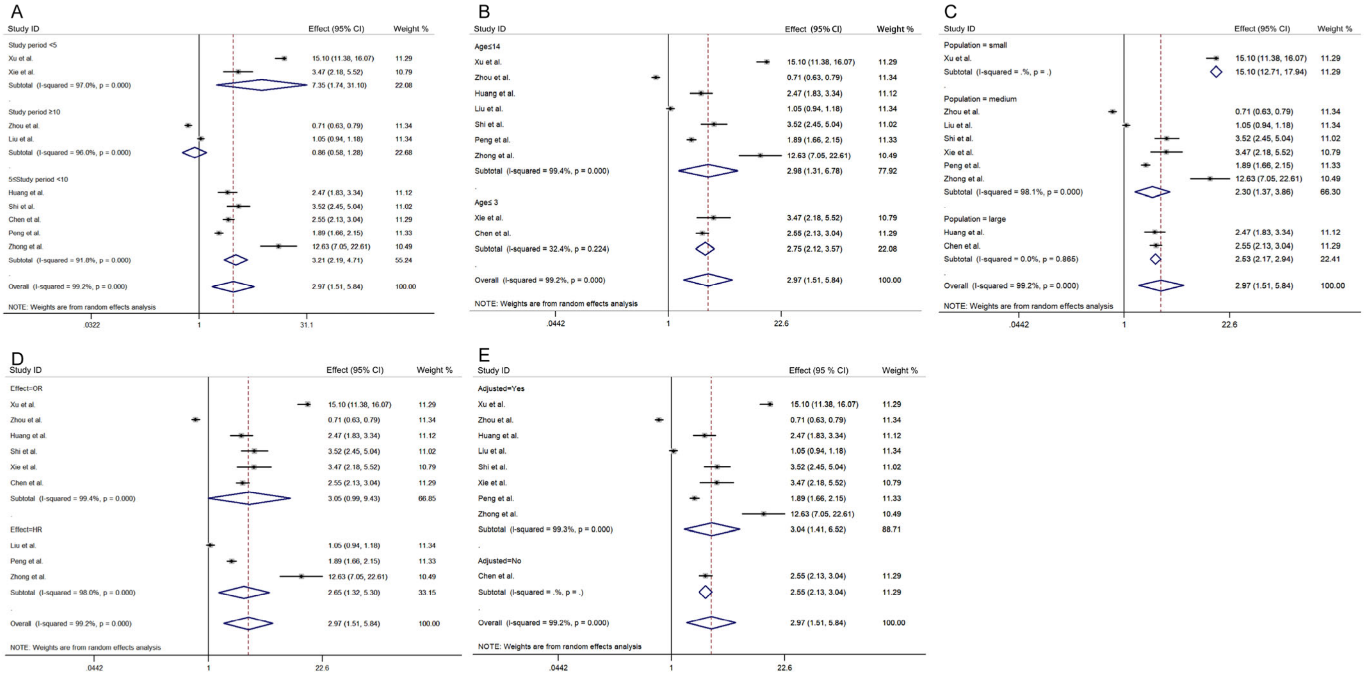

Figure S3 Subgroup analysis of overall effect and 95%CI for age. A (study period); B (age range); C (population); D (effect measure); E (adjustment for confounding variables)

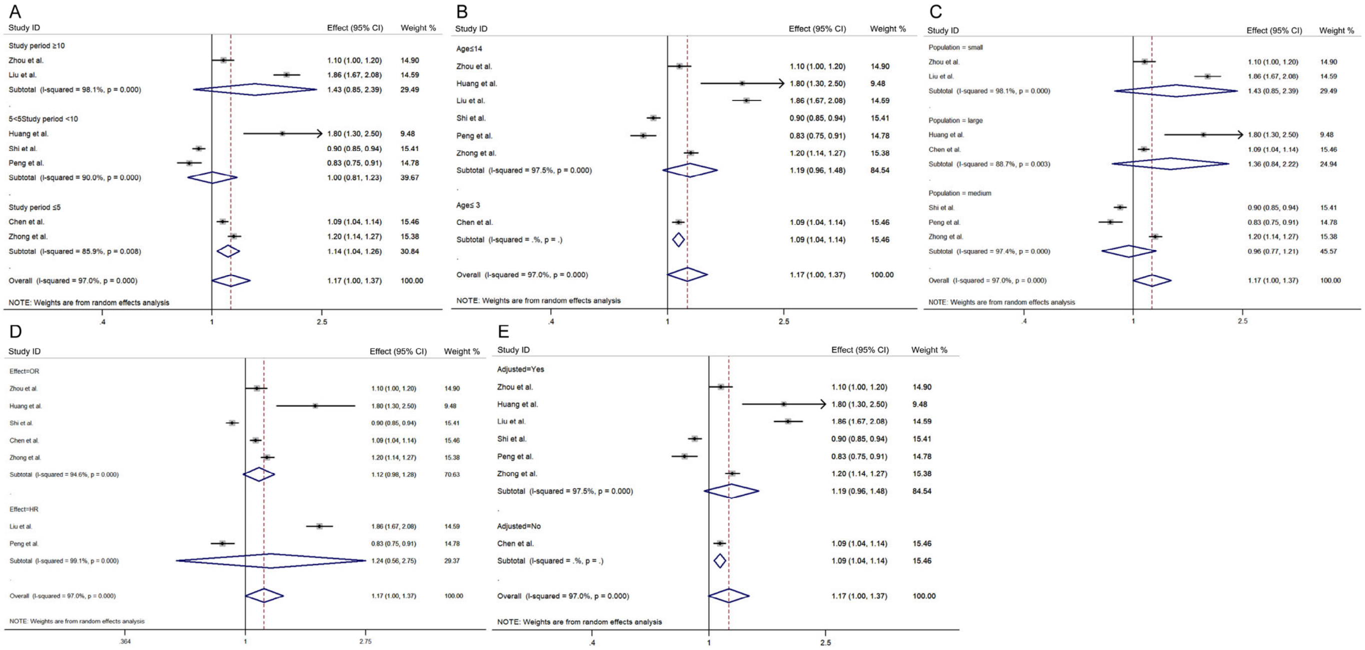

Figure S4 Subgroup analysis of overall effect and 95%CI for residence. A (study period); B (age range); C (population); D (effect measure); E (adjustment for confounding variables)

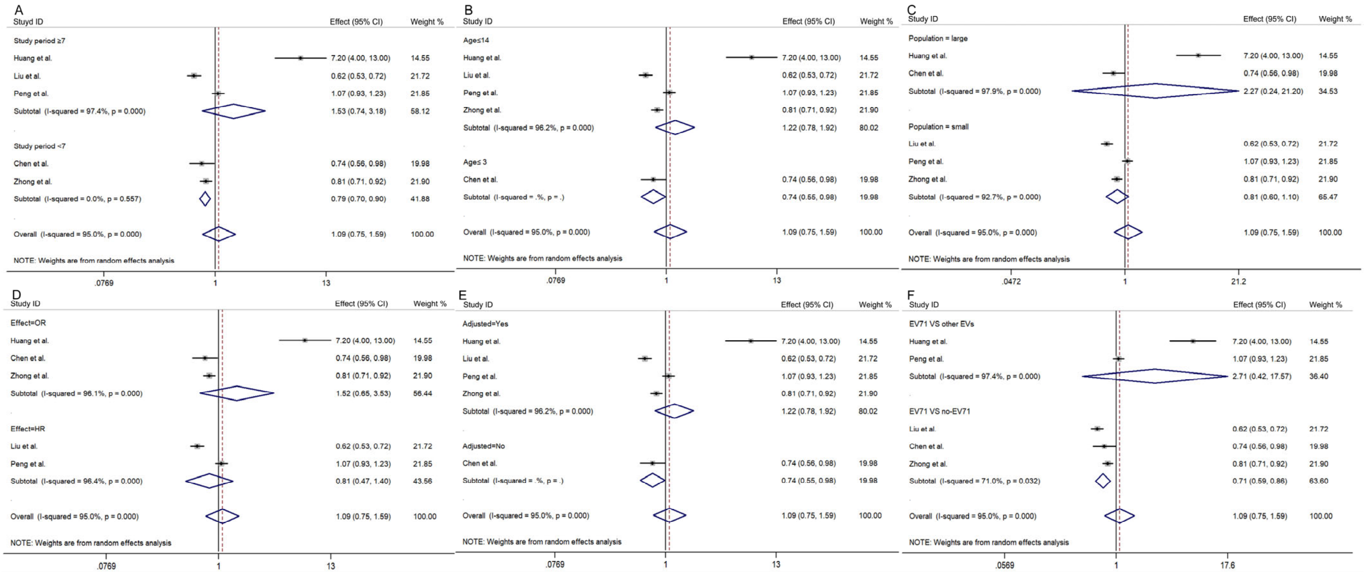

Figure S5 Subgroup analysis of overall effect and 95%CI for pathogens. A (study period); B (age range); C (population); D (effect measure); E (adjustment for confounding variables); F (pathogen variable)

Table S1 Search strategy in different databases.

**Search terms for PubMed (n = 50), until 24 October 2025**
#1 (("Hand Foot"[Title/Abstract] AND "Mouth Disease"[Title/Abstract]) OR ("Hand Foot Mouth Disease"[Title/Abstract]) OR ("Hand Foot"[Title/Abstract] AND "Mouth Disease"[Title/Abstract]) OR HFMD[Title/Abstract])
#2 (Recurrence[Title/Abstract] OR Recurrences[Title/Abstract] OR Relapse[Title/Abstract] OR Relapses[Title/Abstract] OR Recrudescence[Title/Abstract] OR Recrudescences[Title/Abstract] OR "Repeated infection"[Title/Abstract] OR "Repeated onset"[Title/Abstract] OR "Multiple infection"[Title/Abstract])
(#1 AND #2)
**Search terms for Web of Science (n =39), until 24 October 2025**
#1 Topic = ("Hand Foot Mouth Disease" OR "HFMD" OR ("Hand Foot" AND "Mouth Disease"))
#2 Topic = (Recurrence OR Recurrences OR Relapse OR Relapses OR Recrudescence OR "Repeated infection" OR "Repeated onset" OR "Multiple infection")
(#1 AND #2)
**Search terms for Embase (n =17), until 24 October 2025**
#1 'hand foot and mouth disease':ti OR 'hand foot mouth disease':ti OR hfmd:ti OR 'hand foot and mouth':ti
#2 recurren*:ti OR relapse*:ti OR 'repeat* infection*':ti OR reinfect*:ti OR 'multiple episode*':ti OR 'repeated onset':ti OR 'second episode':ti
(#1 AND #2)

Table S3: NOS scores of included studies

First author	NOS	Is the case definition adequate?	Representativeness of the cases	Selection of Controls	Definition of Controls	Comparability	Ascertainment of exposure	Same method of ascertainment for cases and controls	NOn-Response Rate
Xu et al.	6	0	1	1	1	1	1	1	0
Zhou et al.	8	1	1	1	1	2	1	1	0
Huang et al.	9	1	1	1	1	2	1	1	1
Liu et al.	7	1	1	1	1	1	1	1	0
Shi et al.	7	1	1	1	1	1	1	1	0
Xie et al.	7	1	1	1	1	1	1	1	0
Chen et al.	8	1	1	1	1	2	1	1	1
Peng et al.	7	1	1	1	1	1	1	1	0
Zhong et al.	7	1	1	1	1	1	1	1	0

Table S4 Begg'test of the overall RR and 95%CI of different air pollutants

Variable	Begg'test		Egger'test	
	z	P	t	P
Reinfection %	1.88	0.076	-2.01	0.084
Sex	1.25	0.251	1.07	0.32
Age	1.77	0.076	1.59	0.157
Residence	0.6	0.548	0.84	0.437
Pathogens	0.24	0.806	1.24	0.303
Severe	1.02	0.308	-1.68	0.235
